# Implementing Organizational WHP Into Practice: Obstructing Paradoxes in the Alignment and Distribution of Empowerment

**DOI:** 10.3389/fpubh.2020.579197

**Published:** 2020-12-22

**Authors:** Katrin Skagert, Lotta Dellve

**Affiliations:** ^1^Division Materials and Production, Research Institutes of Sweden (RISE), Gothenburg, Sweden; ^2^Department of Sociology and Work Science, University of Gothenburg, Gothenburg, Sweden

**Keywords:** alignment, structural empowerment, distributed leadership, system theory, implementation, health care organizational setting

## Abstract

**Background:** According to policy and theory, there is need for organizational workplace health promotion (WHP) to strengthen working conditions for all employees. However, earlier studies show it is hard to implement in practice. The aim was to critically analyze and identify interacting mechanisms and obstacles behind failures of organizational WHP projects from system perspectives.

**Methods:** A holistic case study was performed, to critically analyze data from an organizational WHP project approach at a public health care organization. The qualitative data was collected over 5 years and included interviews with key actors (*n* = 80), focus groups (*n* = 59 managers), structured observations (*n* = 250 hours), continuous field observations and documents (*n* = 180). Questionnaires to employees (*n* = 2,974) and managers (*n* = 140) was complementing the qualitative-driven mixed method approach.

**Results:** The analysis shows obstructing paradoxes of alignment and distribution of empowerment during the process of implementation into practice. The obstacles were interacting over system levels and were identified as: *Governance by logics of distancing and detaching, No binding regulation of WHP, Separated responsibility of results, Narrow focus on delegated responsibilities, Store-fronting a strategic model, Keeping poor organizational preconditions and support for developments* and *Isolate WHP from other organizational developments*.

**Conclusions:** The following premises can be formulated regarding successful organizational WHP programs. Consider (1) the uncertainty a distributed empowerment to all system levels may create; (2) the distributed impact to define the target and allow broader areas to be included in WHP; and (3) the integration into other development processes and not reducing the organizational WHP to the form of a project.

## Introduction

Empirical studies and theoretical developments relating to successful workplace health promotion (WHP) in organizations highlight the importance of integrated focus on strengthening resources for health and developments at all organizational and work system levels (1–3). The integrated system approach of WHP, which increases empowerment of conditions supporting health and healthy work conditions, is suggested to be more sustainable, but there are limited studies on the more holistic approaches of WHP, such as organizational WHP (4). However, the implementation of such organizational WHP approaches can meet significant barriers between and within system levels (5, 6). Increased knowledge of the interaction of obstacles at each level and between levels can have importance for implementation of WHP projects, i.e., to better recognize and meet barriers to alignment and to distribute mandates for assessing, defining and conducting WHP activities. This study critically analyzes the implementation of a public organization's organizational approach of WHP that failed despite high ambitions. The study contributes to development of WHP theory by highlighting the obstructing paradoxes of distributed influence and learning as a necessary condition for empowerment and managerialism as norm in accountable public organizations (7).

The workplace is one important setting for enhancing health and well-being (8, 9). Organizational WHP considers structural measures with the aim of improving health for all employees (10), e.g., through strengthening working conditions (5), influence and access to resources and support structures in organizations (structural empowerment) (11, 12). Such organizational approaches of WHP have been highlighted from many perspectives. Policies point to the more holistic, system approaches of WHP, i.e., how the work is organized and an employee's ability to influence at work (13, 14). Theories of WHP and organizational change imply the importance of not (only) focusing on the individual but also the system and organization. Also, empirical studies of outcomes have concluded that WHP is most effective and sustainable when organizational levels are approached; when preventive and promotive perspectives combined; and when improvement of health are all integrated with other organizational improvement processes [see e.g., (15–17)]. Earlier studies have reviewed and identified the most important factors for improving workers' health (18–22), and the evidence-based knowledge is quite robust. However, the significant interactions over and between individual, group, and organizational factors are less known. Some studies show a stronger magnitude of risks/resources for the interacting factors than for the single factors (15, 23). Therefore, WHP work based on the knowledge of how to handle interactions across organizational levels is needed and crucial for sustainable developments of employee health. This is also supported by studies showing that managerial work based on actively bridging organizational levels to integrate perspectives have had more success in producing sustainable organizational developments (24–26). Consequently, broader organizational approaches of WHP interventions would generally have a stronger effect than a WHP intervention focusing on single targets. Likewise, WHP at several levels could have a stronger effect than those focusing on one system level.

Nevertheless, WHP interventions most often focus on individual behavioral change rather than workplace change (27–31) even when the identified core challenges are clearly related to organizational conditions (32). Thus, despite the theoretical developments of WHP and global policies, knowledge about effective measures and approaches to improve working conditions is still needed (33–36). This includes knowledge of the central obstructing mechanisms and driving forces that hinder implementation and sustainability of organizational WHP.

Sustainable improvements of work organizations are understood, from a system perspective, as the continuous interaction between dimensions of intentions and the handling of actors, embedded in social and cultural conditions (37, 38). A theoretical framework for organizational WHP based on system theory suggests possible conditions of importance for crafting WHP conditions at each system level and in between (2). The framework integrates the key multi-conditions for WHP sorted into system levels, as well as the managerial work and organizing practices for crafting and bridging WHP across systems and levels. Such system perspectives on WHP focus more holistic approaches of factors, conditions and contexts at different levels: At the workplace in the daily work (micro-level); within the rules, structures, norms, and values of the organization (meso-level); with regard to impacts from the wider organization and society (macro-level) and related to temporal aspects and developments that may start at one level but have implications for all levels (chrono-level). Thus, implementation of organizational WHP requires distribution of empowerment, supporting influence and commitments (39) across systems. In connection to such system perspectives, alignment seems crucial for a stable common understanding of the organizations goals, purpose and vision regarding WHP (40). A functional alignment and distribution of empowerment is necessary (a) vertically so all the members of the organization know what and why a certain behavior is needed to contribute to the common goals of the organization, have mandates and can take actions, (b) horizontally between different work processes or units, and (c) diagonally, where superior executives and strategic management act as role models and synchronize and facilitate the desired actions at the lower hierarchical levels (the operative level) (41). Nevertheless, there is still a lack of knowledge of the mechanisms related to how conditions, drivers and obstacles interact between vertical, horizontal and diagonal levels.

This paper reports experiences from a case study of an implementation of organizational WHP at a medium-sized hospital in Sweden. The aim was to describe the implementation processes and critically analyze interacting mechanisms and obstacles behind failures of organizational WHP projects. Thus, the paper contributes to development of WHP theory by identifying the obstacles at each level involved in the proximal processes hindering distribution of empowerment over system levels.

## Materials and Methods

### Study Design

To analyze global characteristics of a program, the study-design was a holistic case study approach (42). Case study design is recommended when (1) the aim is to understand complex interrelations between the phenomena studied [i.e., the implementation of and organizational WHP and their context (43)]; (2) the research ambition is to analyze ‘thick’ descriptions that represent different perspectives and (3) the researcher has little control over studied events but is interested in naturally occurring variability (44). The case was an implementation project of organizational WHP at a medium-sized hospital in Sweden. The study spans 5 years. Each phase of the implementation (the planning, active and integrated phase) (1), as well as the critical analyses of interacting obstacles, were primarily based on qualitative analyses of data from interviews, observations and documents, and supported by quantitative analysis of questionnaires. Thus, the major theoretical drive was inductive, i.e., a qualitative-driven mixed-method approach (45). The study was approved by the Regional Ethical Review Board (Dnr 433-10).

### Study Setting and the Studied Case

The study took place in Sweden, where occupational health and safety management has been legislated since the late 1800's. The Swedish Work Environment Act (1977:1160) aims to prevent ill-health and accidents at work and achieve a good work environment. The labor market in Sweden has a long tradition of cooperation between employers and employees (i.e., union representatives) and this is also stipulated both in the work environment law and collective labor agreements. Although the workplace is often highlighted as an important arena for enhancing health and well-being, there is no binding regulation regarding workplace health promotion. There are, however, regulations that have shaped, constrained, and/or strengthened the occupational health and safety management, aiming to prevent ill-health rather than promote health.

The initiative for WHP was taken by the county council (macro-level). A steering board of union and employer representatives at the top level decided to take a further step toward putting workplace health promotion into practice. They identified a hospital as a preferred organization for such an implementation initiative and also had initial contact with the research group to study the implementation process. The studied organization (meso-level) was a middle-sized public hospital where process management and continuous improvement of processes had been going on for several years when this study started. The hospital had acute and planned care (including psychiatric care), a total of 800 beds and ~4,500 employees (82% women and 18% men). The hospital management teams' ambition was to implement workplace health promotion, with an organizational approach, and organized an implementation project for this purpose.

### Data Collection

The holistic case study used a qualitative-driven mixed method approach for data-collection. For the purpose of the study, the qualitative data from interviews, focus groups, documents and notes from observation was the main source of data. Data from structured observation and questionnaires was complementing to provide broader descriptions and general views of and conditions for organizational WHP. The materials included in the holistic case study are described below:

*Documents* From year one (Y1) to year three (Y3), the implementation process was followed in the hospital's WHP process plans, management protocols, interviews and field notes. Data collection included all meeting protocols from the hospital top management (*n* = 60, ~250 pages) and from the three clinical divisions into which the care was organized (*n* = 120, ~350 pages).

*Individual interviews* In-depth interviews were conducted (Y1-Y3) with key functions in the implementation process (*n* = 5) and line managers (*n* = 12). The interviews focused the implementation processes and important interacting conditions. The interviews were taped and additional notes were made. In order to better understand governance approaches, county council politicians (*n* = 45, Y1 and Y5) and key functions for organizational developments (*n* = 18) were interviewed about strategies to support improvements in hospital organizations. The majority of the interviews were transcribed; with the remainder careful notes were taken.

*Focus-groups* All first- and second-line managers and a strategic sample of employees from different professions and wards were invited to focus-group interviews, to discuss working conditions, their WHP approaches and the organizational WHP program. Altogether 59 managers participated in nine focus groups (Y1) and 68 employees participated in 12 focus groups (Y3). All focus-groups were transcribed.

*Observations* were made of work-place meetings (*n* = 9, Y2-Y3) and top-management meeting (*n* = 7, Y2-Y3). For the purpose of the study, continuous field notes were also taken from ongoing contacts on site with managers at different levels and key-functions for implementation (Y1-Y5). Additionally, the continuous contact through e-mails, meetings and phone with leaders of the WHP project (about 3–12 contacts per month, Y1-Y3) was also used as data. Field notes and theoretical memos were written throughout the research process.

*Structured observations* In order to further assess signs of implementation in practice, 12 randomly selected first-line managers were shadowed regarding their work and time allocated to development work and other tasks, contacts and places of work. The observations were directly coded through a computerized structured observation scheme. Thus, the time used for each activity was observed by a researcher and directly registered using a computer program (46).

*Questionnaires* All first line managers were invited to answer a questionnaire, distributed through emails (*n* = 140, response rate 75%, Y2). The responding managers (*n* = 105) mean age were 49 (md = 51, range 25–63 years of age). Most (85%, *n* = 89) worked full-time as managers, other part of their working-time. The following variables were analyzed for the purpose of the present study: leadership approaches and support through superior manager (47). All employees were also invited to answer a questionnaire (*n* = 2,974, Y3, response rate 65%). For the purpose of the study, items of improvement work, improvement of quality of care, working conditions and efficiency were included in the analysis.

### Analysis

The transcribed interviews, focus groups, field notes and documents (the qualitative raw data) were analyzed stepwise coded with manifest and latent codes in line with content analyses (48). First, descriptive qualitative analysis of the qualitative data were made sentence by sentence, to describe the chronological time and activities in the implementation project at the hospital (manifest coding of content). The second step in the qualitative analysis (latent coding) focused contextual factors and conditions of importance for the implementation process. The analytic latent coding was conducted based on system theory perspectives of organizational WHP and alignment over macro-, meso-, micro and chrono-levels. The result from the manifest coding resulted in descriptions of failures at several levels that were observed to be connected. Therefore, we chose to focus the latent coding on a critical perspective of the central obstacles for the development of organizational WHP, i.e., approaches, conditions and mechanisms that bridged system levels.

The complementing quantitative data were used to serve as examples and add additional perspectives to the qualitative analysis. Structured observations of managers were analyzed with descriptive statistics of time used on different activities. Descriptive analyses were conducted with questionnaire data. Prevalence ratios was also calculated, with data from employee questionnaire, for assessment of statistical differences of proportions (PR95CI).

## Results

The first result section describes the phases and central conditions of the implementation process. The second section presents the main categories from the critical analysis of proximal processes and key conditions for alignment and distribution over and within system levels.

### Case Description: Phases and Conditions for Implementation

**Figure 1:**
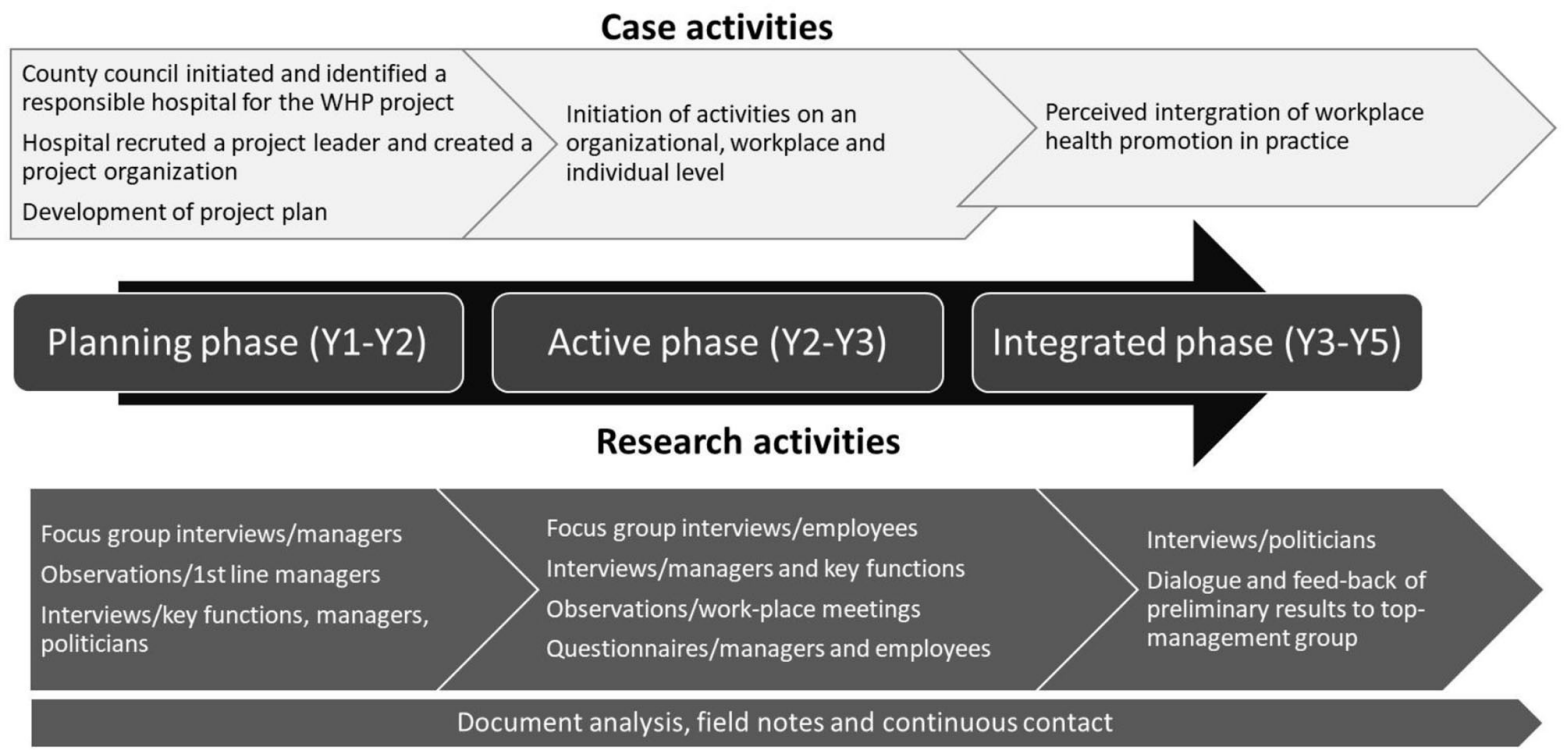
Overall view of the case activities in the WHP project and the research activities.

First, the implementation process in terms of the initiative for the project, the organizing and the activities are described in three partly overlapping phases: the planning phase, the active phase and the integrated (or not integrated) phase. Figure 1 and Table 1 lists the activities and to what extent they were performed.

**Table 1:** Overall view of the implementation plan activities and the degree to which activities were performed.

**Planned activities on the organizational level**	**Performed and/or implemented**
Clarify and define the WHP perspective in management strategies and goals, governing documents, and follow ups	Done in the active phase but disappeared in the (not) integrated phase
Integrate WHP competence development with ordinary process development	Partly done in the active phase but disappeared in the (not) integrated phase
Clarify cost and cons of workplace health	Not done or integrated in any phase
Develop support and guidelines for systematic workplace health and work environment management	Done in the active phase, no clear integration
Perform education in WHP leadership	Not done in any phase
Create a system to bring leaders and employees up to date with stress related health problems	Not done in any phase
**Activities on the workplace level**	**Performed and/or implemented**
Development of a WHP dialog material with different themes to be distributed to all workplaces. The objective was for it to be used at workplace meetings in order to assess areas and conditions to strengthen	Done and partly integrated in active phase
**Activities on the individual level**	**Performed and/or implemented**
Develop guidelines and health promotion advice, for example to night shift workers	Not done in active or integrated phase
Offer a wide range of wellness benefits for the employees	Not done in active or integrated phase

The *planning phase* lasted about one and a half year (Y1-Y2). The initiative to implement a WHP perspective in the organization came from a steering board with both union representatives and employers alongside the county council. One division in the county council was appointed to be a test arena for the implementation. A project organization was planned and the responsibility for the project was placed at the hospital's human resource (HR) unit by the hospital director. The project team consisted of a work environment strategist, two union representatives, one person who ordinarily was responsible for patient-related health promotion work, and an externally recruited project leader with a master's degree in public health. There was no project plan in place when the project leader was recruited, so her first task was to immediately start to write a project plan. It was an ambitious plan, based on a system theoretical holistic perspective and existing evidence on what distinguishes a WHP organization. The hospital's management team approved the project plan with goals and activities at the end of year one (the planning phase). The project plan had goals and activities on three (organizational, workplace and individual) levels. The overall goals were:

- Implement a WHP perspective in strategic management and governing documents
- Strengthen employee influence and participation in assessment of defining areas and resources to strengthen and open communication climate
- Enable health-promoting choices for the individual (employee)

According to the project plan, the *active phase* started at year two (Y2-Y3). When concrete activities were due to take place and be performed in the organization, several were rejected by the top management team with reference to economy or timing (see Table 1). Some of the activities seemed to disappear due to unclear communication and distribution of responsibility for the activities or mandates to take decisions. The top management's lack of responsibility and engagement was expressed in the interviews as an explanation for the uncertainty.

“One of the most important, if not the most important, things when you run this type of change process is to have the highest management fully engaged and I do not feel that the project has that/…/ but I think you have to decide in the hospital management whether you should seriously do this work at present or if you should actually put it on ice.” – interview with person within the project team

Managers and employees also seemed to have different views on health. The project plan was based on the system theoretical view of health and activities mainly focused on organizational conditions, while the more traditional WHP focus on lifestyle activities was more widespread among operative managers and employees. In addition, at the organizational structural level, there were different views on whether the implementation was an HR-related process or an organizational improvement process. Altogether, this contributed to a lack of alignment and accountability of measures at different levels in the organization. The various views and expectations of the project collided, and the first project leader felt caught in the middle and resigned after 1.5 years. The next project leader was a HR specialist with more than 30 years of experience at the hospital. She also resigned after 1 year and was replaced with an externally recruited HR specialist who also resigned about a year later.

According to the project plan, the *integrated phase* started at year three. To assess implications from the WHP project, all protocols from management teams at hospital- and clinical level were analyzed regarding their content. These protocols clearly indicated only one-way information from the WHP project team. Further, all initiatives for planned activities suggested by the WHP group or operative management to fulfill policy goals in practice were not approved by the hospital management group. For example, health coaches' desire to meet and share experiences and tools between organizational units was rejected. Despite the low activity regarding WHP, the law-regulated and prescribed health preventive measures seemed to function well at all levels. All agreed plans regarding prevention were followed by activities and follow-up regarding sick leave, work-related diseases and work-related injuries.

**Figure 2:**
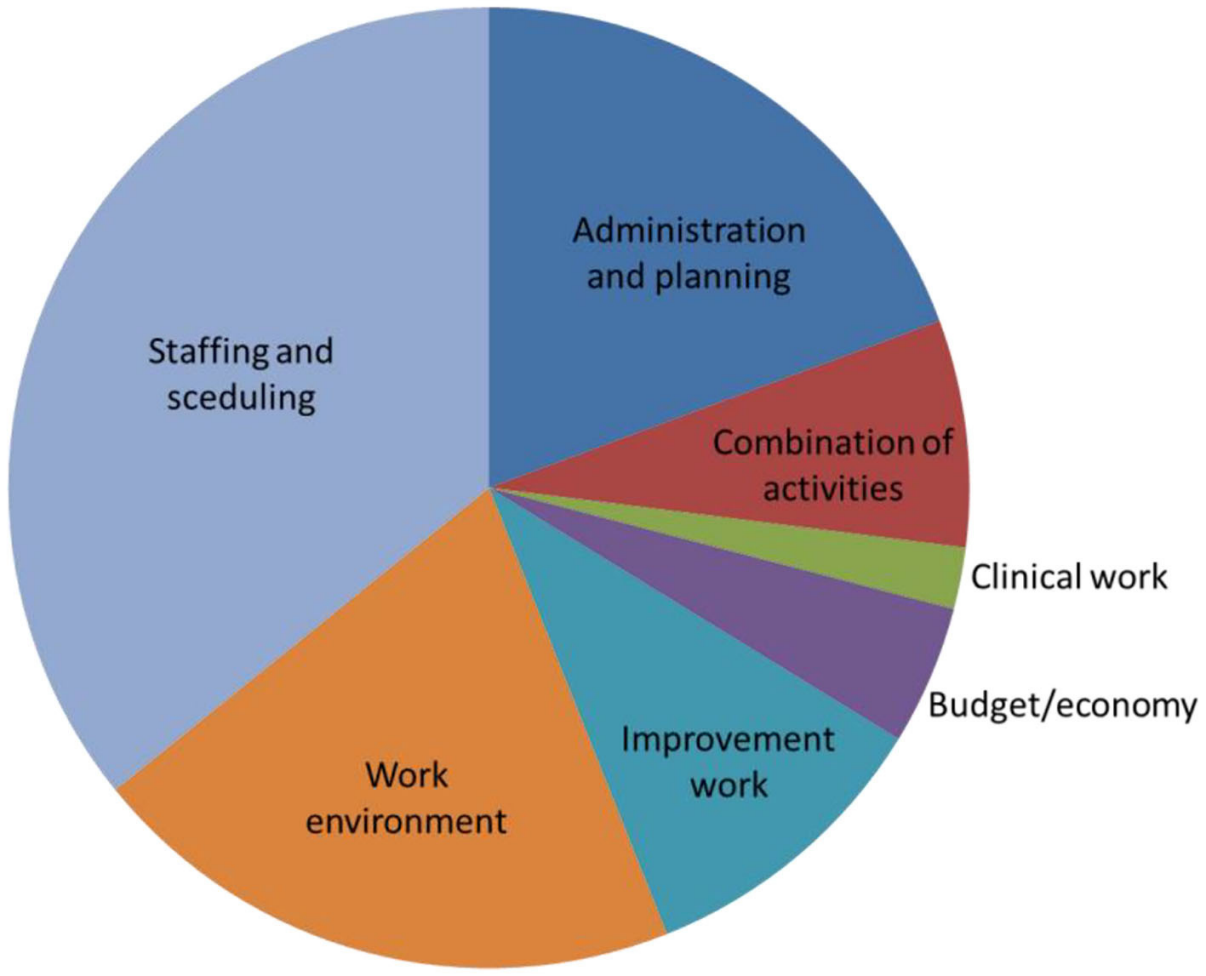
Observed time-use among first-line managers.

Second, conditions of importance for active work with WHP at operative levels were assessed. The managers, both first- and second-line, claimed that the support of communication flow between organizational levels and the support of empowerment and participation of subordinates were given the highest priority. Interviews, questionnaires and observations of the first-line managers showed their time conflicts related to allocation of time for the development work. The observation showed that work with developments happened during scheduled meetings (3% of their total working time). Little time was also allocated to communication between superiors and subordinates. First-line managers were on average communicating face-to-face with their own manager for 0.5% of their total working time and 10% met their manager every day. Most of the time (67%), first-line managers were working at their office alone. Most of their time was allocated to administration or staffing challenges to solve immediate problems in the clinical work (Figure 2). The majority (87%) of the first line managers assesses, through the questionnaire, that their leadership approach could be characterized as participative. And, that they gave their employees opportunities to have influence over the development work at the unit. The majority (82%) also rated that they often discussed challenges in improvement work with their own manager. However, only 12% met their own manager every day, to discuss challenges. One third (28%) met their own manager a few times per week or month (32%) and 23% even lesser.

Despite managers having little time for aligning the WHP program between strategic and operative levels, the improvement work at operative level was observed as having a high degree of dialogue, participation and influence between the employees at several units. At operative units working more actively with the improvement work, strengthened working conditions (PR95% CI 1.32 [1.25–1.39]), and also improved quality of care (PR95% CI 1.19 [1.15–1.22]) and efficiency (PR95% CI 1.58 [1.50–1.67]) was observed compared to units working less active with improvement work (from analysis of employee questionnaires).

### Obstructing Paradoxes for Alignment of Organizational WHP and Distribution of Empowerment

Here, the result of the critical analysis of the lack of alignment and distribution of structural empowerment for WHP across organizational levels are presented. The approaches and decisions are contradicting and interacting (paradoxes) across system-levels and thus obstructing alignment for organizational WHP and distribution of empowerment. The key obstacles are placed on the system level where they were based (Table 2).

**Table 2:** Key obstacles for alignment and distribution of WHP across organizational levels.

**Macro-level obstacles**	**Meso-level obstacles**	**Micro-level obstacles**	**Chrono-level obstacles**
*No binding regulation of WHP: talking but little action* *Governance by logics of distancing and detaching: separated responsibility of results*	*Focus clearly delegated responsibilities* - WHP reduced to prevention *Creating and store-fronting a strategic model* - Disenabling bureaucracy - Dumping implementation on HR and a group with little power - Persisting complex systems hindering follow-up	*General poor organizational preconditions and support for development* - Administrative and staffing load on operative managers - Delegated and detached responsibilities for WHP at operative levels	WHP activities isolated from development of clinical core processes - Bad timingNon-bridging over levels and perspectives - Isolated good examples

### Macro-Level Obstacles

The governance approaches of the studied county council (macro-level) were characterized by *Logics of distancing and detaching*. This implied deciding and providing preconditions but having a detached approach, with delegation, separation of responsibilities of results and a filtering of communication between organizational levels and functions. The governance approach was observed to bridge system levels; i.e., the logics of distancing and detaching were propagated as the valid management approach for public organizations. The macro-level conditions were also characterized by the non-binding regulation of WHP, which meant a lot of talking about the importance of organizational WHP was not followed by action.

### Meso-Level Obstacles

Obstacles in hospital top-management (the meso-level) were connected to the macro-level governance. The following meso-level obstacles were identified:

*Focus on the clearly delegated responsibilities*. The decision to implement an organizational WHP project with no demands for results was taken at the county council level. The hospital management team focused on their clearly regulated and delegated responsibilities. Reasons for these choices were the macro-level lack of regulation and demands of WHP, the hospital management teams' lack of genuine interest in WHP, and the lack of competence to handle conflicting organizational interests. Thus, their approach implied a management focus that *reduced WHP to the regulated health prevention* measures, which were applied and reasonably well-managed and negotiated at all organizational levels. At the same time, the organizational WHP was strategically vaguely managed by rhetoric and a store-fronting policy model.

*Structure and store-fronting a planned policy model*. To fulfill the agreed WHP assignment, the management group decided a strategic plan and policy model for the WHP project based on best available evidence and with plans at meso- and micro system levels. This document was store-fronted upwards to county council levels to legitimize their accomplishment of the WHP project. This implied no further questioning from the county council level. Thereafter, a number of obstacles for the activity and integration phase were observed: (a) placing the project at the HR unit with a loose connection to the clinical core process and daily work practice, (b) dumping implementation on a group with little or no within-organizational power, (c) disenabling bureaucracy for distributing mandates, and (d) allowing complex systems that were hindering follow-ups.

An important obstacle was dumping the responsibility for implementation on a “satellite group” that was loosely anchored in the organization and had little power. The placement of WHP with a small group within the HR function served to isolate the WHP project from other organizational developments led by other organizational functions and spread over the hospital. The recruitment of a project leader with little earlier practical experience of hospital organizations was another approach to limit the influence of the group. Thus, the group and the project leader had difficulties in raising interest from clinical departments and supportive resources from staff functions. Instead, they further developed the written plans and handbooks despite their major difficulties in anchoring these at operative levels. Also at this level, the non-binding regulations regarding WHP meant that there was much talk and policy about intentions, interests and values but little action and prioritizing to fulfill those intentions.

The general governance approach of the county council, characterized by logics of distancing and detaching, acknowledged a detached approach of managing and organizing the WHP project, with delegation, separation of responsibilities and filtering of communication over organizational levels and functions. This seemed to hinder the operative managers, employees and professionals to have an overview of, engage with and exert influence over the WHP work. These approaches were in line with the governance of distancing and detaching, and can be characterized as measures of *disenabling bureaucracy –* a designed organizational structure that decreases the influence and control outside the management group while also delegating responsibility for the accomplishment of the required operative tasks. The disenabling bureaucracy hindered empowerment conditions through formal organizational structures and social formations of communities. The disenabling bureaucracy was observed as related to difficulties in fulfilling the goals of the strategic model in practice due to lack of mutual interest in the focused issue, economic resources, time and functional support. Further, the design of *non-bridging independent systems of the organizational structure* guaranteed the detached approach. Three main systems of register-based information were used in parallel and hindered follow-up regarding both accomplishment of responsibility and results. One collected information about economic issues, one about sick-leave and other vacancies and another about salary. None of these had the same picture of the organizational structure, including information about managers in charge that was shown on the hospital website. None of the systems covered all employees or followed the same organization structure.

### Micro-Level Obstacles

The analyses identified hindering conditions in terms of *poor organizational preconditions* that prevented operative managers and other functions from participating in, engaging with and taking wider action regarding WHP. In this case the setting was characterized by high administrative and staffing load as well as dumped responsibilities without mandates for WHP at operative levels.

A heavy *administrative and staffing load* was placed on operative managers, for example through a large span of control, delegated responsibility of administrative duties, and no organizational support in staffing. The *dumped* (*delegated*) *responsibilities for WHP* at operative levels implied no organizational support or even interest from staff resources or the management team. The lack of support was expressed by operative managers and WHP coaches and also observed in protocols where no general organizational support for local or central WHP initiatives was accepted. Some of the managers also expressed poor competence in the WHP area and experienced little support from WHP coaches at operative level. Instead, employees with specific interest in wellness and fitness activities took responsibility for involving colleagues in such individual-focused health-promoting life-style activities.

### Chrono-Level Obstacles for Development

The chrono-level encompasses the dimension of times, developments and trends of interests of WHP and work environment issues. Important conditions for developments are aligning the WHP activities over organizational levels and also integrating the perspectives of effectiveness, quality, and the work environment. Here, an important initial obstacle was to isolate the WHP activities from the development of clinical core processes by placing WHP within HR and isolated to a satellite group. Then, the *timing* of each WHP initiative was bad and the other obstacles observed and described above were successful in hindering the bridging and alignment of WHP activities and initiatives over organizational levels and units. This implied that there were isolated good examples of WHP that were not spread. Thus, the co-workers did not in general observe any WHP activities at the hospital.

## Discussion

This case study aimed to describe and critically analyze the implementation process of organizational WHP projects. The key result describes the activities in the planning, active and integrated (or not integrated) phases of a WHP project and the analysis shows obstructing paradoxes of alignment and distribution of empowerment during the process of implementation into practice. Thus, the approaches and decisions were contradicting and interacting (paradoxes) across system-levels and thus obstructing alignment for organizational WHP and distribution of empowerment. The important obstacles were identified as: *Governance by logics of distancing and detaching, No binding regulation of WHP, Separated responsibility of results, Narrow focus on delegated responsibilities, Store-fronting a strategic model, Keeping poor organizational preconditions and support for developments* and *Isolate WHP from other organizational developments*.

To sustain organizational change, the WHP project needs to be integrated into work practice (1) in all system levels of an organization (2). This was also basically the stated objective in the studied organization's WHP program. Yet it failed to be integrated. In line with Rojatz et al. (6), obstacles (or barriers) was found at contextual, organizational, intervention, implementer, and participant level in the different phases. The result of the analysis identified a number of key obstacles at all system levels that “curtailed” subordinates' mandates and structural empowerment as well as the possibility for follow-up within the organization. In the following text, we will try to highlight and problematize proximal processes of importance which can contribute to theoretical developments of frameworks for implementing organizational WHP.

Firstly, organizational WHP programs need to *consider the uncertainty a true distributed empowerment to all system levels may create*, and also the variety of defensive mechanisms that are mobilized to curtail insight and influence over system levels, in terms of: managerialism, bureaucratism (7) and separated systems for documentation and follow-up (49). These increase the gap of knowledge and practice (alignment) between the organization's strategic and operative levels and is mainly described in large public organizations. The macro-level strategies of county council politicians can be understood from the nature of their work, i.e., being based on a high degree of ambiguity, inherent conflicts and uncertainties which often result in avoidance and compromise in trying to balance multiple components and achieve different organizational goals. Nevertheless, the logics of governance seemed to have significant impact on the improvement work across organizational levels. Earlier studies have contrasted local logics of governance strategies (50) and showed higher work engagement among employees over time in more practice-oriented servant governance compared to the detached and upward-focused logics of governance identified in the studied county council (41). In the present study the passively controlling governance was related to top-management's active store-fronting of the program, which was absent within the organization. This is in line with Alvesson's (51) critical conceptualizing of the “triumphs of emptiness,” when management ideas of grandiose change occur without actions at operative levels, and the identification by MacBeath et al. (7) of empty, controlling managerialism as the norm for organizational accountability in public organizations. Consequently, WHP programs would benefit from downward-focused servant leadership, with sincere interest in serving changes at floor through aligning influence and distribution of empowerment in a downward-directed manner to subordinates (26).

Secondly, organizational WHP programs need to *consider the distributed impact to define the target and allow broader areas* to be included in WHP. To have relevance, the assessment and prioritizing of WHP areas and conditions to strengthen must be defined at each system level. This requires a great deal of freedom from normative assumptions of what is “the right WHP.” In the analyzed case, the county council conducted a problem analysis before the project started that was based on earlier research (52), and came to the conclusion that individual-oriented measures only reached a limited amount of the employees and most often those who already had a health-promoting life style. Thus, the project plan aimed to mainly improve organizational conditions for WHP. However, the problem analysis and resulting project plan was distributed within the organization and not further anchored to the local context that had other views of what was needed. Further, previous studies of organizational improvement of working conditions have highlighted the importance of actions being adapted to the problems that really exist and being based on a clear problem analysis that has a broader involvement (31, 35, 53–55). Nevertheless, the concept of problem analysis might be ambiguous to use in a WHP context since it derives from pathogenic rather than salutogenic thinking. Experience showed, however, that when the strengthening activities really started, they often developed into more organizational WHP activities.

The current study is also an example of the contradictions regarding distribution of influence in defining organizational WHP activities: All suggested activities were rejected by top management. Thus, employees were “allowed” to make efforts to improve WHP as long as it didn't have impact on ordinary management and distribution of work. Thus, the case gives a description of poor alignment where the strategy, structure, and culture were not combined to create a “synergistic whole” (56). Instead, the dysfunctional interactions between and within the organization's levels became apparent. This can be a reason for poor sustainability of WHP (40) and highlights the need for continuous critical thinking of structural organizational power dimensions during an organizational WHP project. Theories of empowerment touch the “power” field and need to be complemented with theories of structural power relations involving both formal and informal power (11, 12). This may also yield results from WHP projects as structural empowerment is strongly correlated to health dimensions such as organizational commitment and psychological empowerment in public health care organizations (39). Thus, considering structural empowerment in organizational WHP program support increased effective workplace culture and organizational performance.

Thirdly, organizational WHP programs need to be integrated in other development processes (here: clinical improvements) and cannot be reduced to the form of a project. This WHP project had a well-defined plan according activities to all levels in the organization. However, a plan or strategic policy was not a guarantee of actual implementation of workplace health promotion into practice. The linear idea of implementing work health promotion through activities on different organizational levels might not be useful when it comes to this kind of “zone of complexity” (1). The obstacles for implementation of WHP in the present study were obvious at the meso-levels but related to the macro-level's detachment of responsibility for the results. The timing and lack of integration with the core business made it almost impossible for implementation at the meso- and micro levels. Nevertheless, when WHP was integrated with increased quality of care and effectiveness, there was significantly higher activity and improved working conditions.

The WHP plan included activities of creating a system to bring leaders and employees up to date with stress related health problems. Those activities did not seem to be implemented, still mandatory preventive measures (regulated by the Swedish Work Environment Act) seemed to function both at meso- and micro levels. There is robustly research of the job demands-resources model (57), where job demands can cause burnout and job resources (contribute to work engagement and well-being (58). Due to evidence, The Swedish Work Environment Act has been strengthened lately regarding the employers responsibility to prevent imbalance between job demands and resources. One practical implication of this might be that responsibility and authority for WHP will be closer to core business, and not to a strategic HR department which may increase the distance between WHP and the core business. These results are in line with the findings of Astnell et al. (59), who showed WHP activities increased when integrated with quality improvement work.

Dedicated engagement from top management is crucial for allocating resources in terms of time and competence (49). The allocation of time and priority was not the problem in this case from top-management's perspective. From the observations of operative managers, however, it was clear that little time was allocated to improvement work. Implementation of workplace health promotion into practice seems to have its own challenges in terms of taking abstract visions and strategies and putting them into practice (2). Instead, theory may contribute to a vicious circle between preconditions when implementing WHP and the effects of it. For example, this studied organization had a goal to increase employee influence but did not include participation in the planning phase. That, and the different views of health and health promotion, contributed to the gap between the strategic plan and the core care business.

### Methodological Considerations and Limitations of the Study

The strength of the study is the 5-year long-term follow-up, the use of many measures for of data collection, from key actors representing many different views of and conditions for organizational WHP. The analysis would not come to the same conclusion if only one source of data were being used or the follow-up was shorter. The development of the project and the improvements were shaped and reshaped through contextual factors. The generalizability of the result is therefore not applicable and possible to replicate. Yet, the results may have transferability to similar contexts. Further, the results contribute to the theory frame of organizational WHP and points to the importance of considering broader methods for follow-up than merely chronological baseline-follow-up design. However, the broad descriptions of this article can also be seen as a limitation as results from in-depth analysis at individual and workplace levels was not included. Such in-depth analyses of individuals' and work-group perspectives are given in two thesis (46, 60). Another weakness is the single case-study design. An additional limitation, and at the same time a result of the study, is the lack of organizational follow-up data. One of the projects activities aimed to clarify costs and cons of workplace health. Data related to economic issues, quality of care and staff-related data such as short- and long-term sick-leave was collected in different systems and non-comparable organizational units. This makes it difficult for organizations to evaluate their own efforts to improve employee health.

## Conclusions

This study shows that a well-performed plan for organizational WHP is no guarantee for actual implementation of WHP into practice. The linear idea of implementing WHP in activities on different organizational levels might not be useful when it comes to complex public organizations. Organizational WHP should rather be evolved through continuous improvements related to improvements of core business. The conscious and continuous efforts to improve how work is organized to enable employee influence in the organization should not be underestimated. The following premises can be formulated regarding sustainable organizational WHP programs. (1) *Consider the uncertainty a distributed empowerment to all system levels may create;* (2) *Consider the distributed impact to define the target and allow broader areas* to be included in WHP; and (3) *Consider the integration into other development processes* and do not reduce the organizational WHP to the form of a project.

## Data Availability Statement

Due to the ethical principals, the researchers are responsible for storing data for 10 years. The data supporting the conclusions of this article will be made available by the authors for as long as that.

## Ethics Statement

The studies were reviewed and approved by The Regional Ethical Review Board at Sahlgrenska Academy, Dnr 433-10. The participants provided their written informed consent to participate in this study.

## Author Contributions

KS and LD truly collaborated in design of the work, data collection, data analysis and interpretation, drafting the article, critical revision of the article, and final approval of the version to be published.

## Conflict of Interest

The authors declare that the research was conducted in the absence of any commercial or financial relationships that could be construed as a potential conflict of interest.
